# Indications, clinical outcome and survival of rotating hinge total knee arthroplasty in a retrospective study of 63 primary and revision cases

**DOI:** 10.1007/s00590-022-03349-1

**Published:** 2022-08-22

**Authors:** Andreas Hecker, Hans-Jürg A. Pütz, Sebastian Wangler, Sophie C. Eberlein, Frank M. Klenke

**Affiliations:** Department of Orthopaedic Surgery and Traumatology, Inselspital, Bern University Hospital, University of Bern, Freiburgstrasse 4, 3010 Bern, Switzerland

**Keywords:** Knee, Arthroplasty, Revision, Cause, Outcome

## Abstract

**Purpose:**

The purpose of this study is to report and compare outcome data of both primary and revision cases using a rotating hinge knee (RHK) implant.

**Methods:**

This study retrospectively analyzed 63 cases (19 primary, 44 revisions) at a mean follow-up of 34 ± 8 months after RHK implantation. Outcome parameters were stability, range of motion (ROM), loosening, Hospital of Special Surgery Score (HSS), Knee Society Score (KSS), Oxford Knee Score (OKS), EQ-5D-3L, and Visual Analog Scale (VAS) for overall function. Revision rates and implant survival are reported.

**Results:**

Eleven percent showed medio-lateral instability < 5 mm, a mean ROM of 115° ± 17° and radiologic loosening occurred in 8% (2% symptomatic). PROMS showed the following results: HSS 79 ± 18, KSS 78 ± 27, OKS 26 ± 10, EQ-5D index 0.741 ± 0.233 and VAS 70 ± 20. Primary cases revealed better outcomes in HHS (*p* = .035) and OKS (*p* = 0.047). KSS, EQ-5D index and VAS did not differ between primary and revision cases (*p* = 0.070; *p* = 0.377; *p* = 0.117). Revision rate was 6.3% with an implant survival of 96.8%.

**Conclusions:**

RHK arthroplasty can be performed with good clinical outcome and low revision rate in revision and complex primary cases. RHK is an option in cases where standard arthroplasty and even implants with a higher degree of constraint have reached their limits.

**Level of Evidence:**

Level III, retrospective cohort study.

## Introduction

Hinged or highly constrained knee arthroplasty is a valuable treatment option for surgical salvage in situations where a sufficient joint-stability cannot be accomplished with conventional, unconstrained implants.

Especially in revision surgery with ligamentous laxity and instability, and/or excessive bone loss, or extensor mechanism impairment an increased level of implant constraint may be required. However, this implant type is also being used in primary TKA, where it may offer a viable surgical alternative in cases of severe degenerative and inflammatory arthritis with extreme deformity, bone loss and/or ligamentous insufficiency. [2, 22]

Rotating hinge implants (RHI) were introduced in the late 1970s with the aim to prevent the complications (mainly mechanical failure) of fixed hinge implants [21, 24]. The most significant improvement in the design of these prostheses is the ability to rotate, the introduction of metal wedge augmentation and modular fluted stems with variable offset, achieving better alignment and press-fit fixation. Due to these mechanical improvements, complications of hinged TKA decreased dramatically [14]. Nevertheless, heterogeneous results and frequency of complications have been reported [14, 23]. A short-term (1–5 years) survival of up to 92% and mid-term (6–10 years) survival of up to 82% has been shown. The most common reasons for revision surgery were infection, aseptic loosening and peri-prosthetic fractures [1, 7, 8]. When used in revision surgery, the main indications for an RHI were aseptic loosening of prior TKA, bone loss, ligamentous instability, peri-prosthetic fractures, and infection. [24] When used in primary cases the main indications where ligamentous insufficiency, bone loss, and gross joint destruction [12].

Due to the heterogenty of outcome data regarding this kind of implant the objective of the current study is to report the indications, clinical outcomes, and survival associated with the use of RHI for primary and revision cases.

## Material and methods

This study represents a single-center retrospective study assessing the outcome after implantation of a Rotating Hinge Knee (RHK) arthroplasty (GMK Hinge, Medacta international, Castel San Pietro, Switzerland) performed at our institution between December 2015 and August 2018. The cantonal ethics committee approved the study.

Inclusion criteria were primary and revision total knee arthroplasties with the use of the above-mentioned implant, performed for any reason with available outcome scores at a follow-up of a minimum of two years. [5, 6, 10] Exclusion criteria were the usage of other implants, refusal of participation and incomplete final follow-up data.

In total, 63 RHKs (right 38, left 25) in 63 patients (37 female, 26 male) were evaluated. The mean age at the index-surgery (RHK implantation) was 69 ± 9 years. The mean follow-up was 34 ± 8 months. Complete demographic data is given in Table 2.

**Fig. 1:**
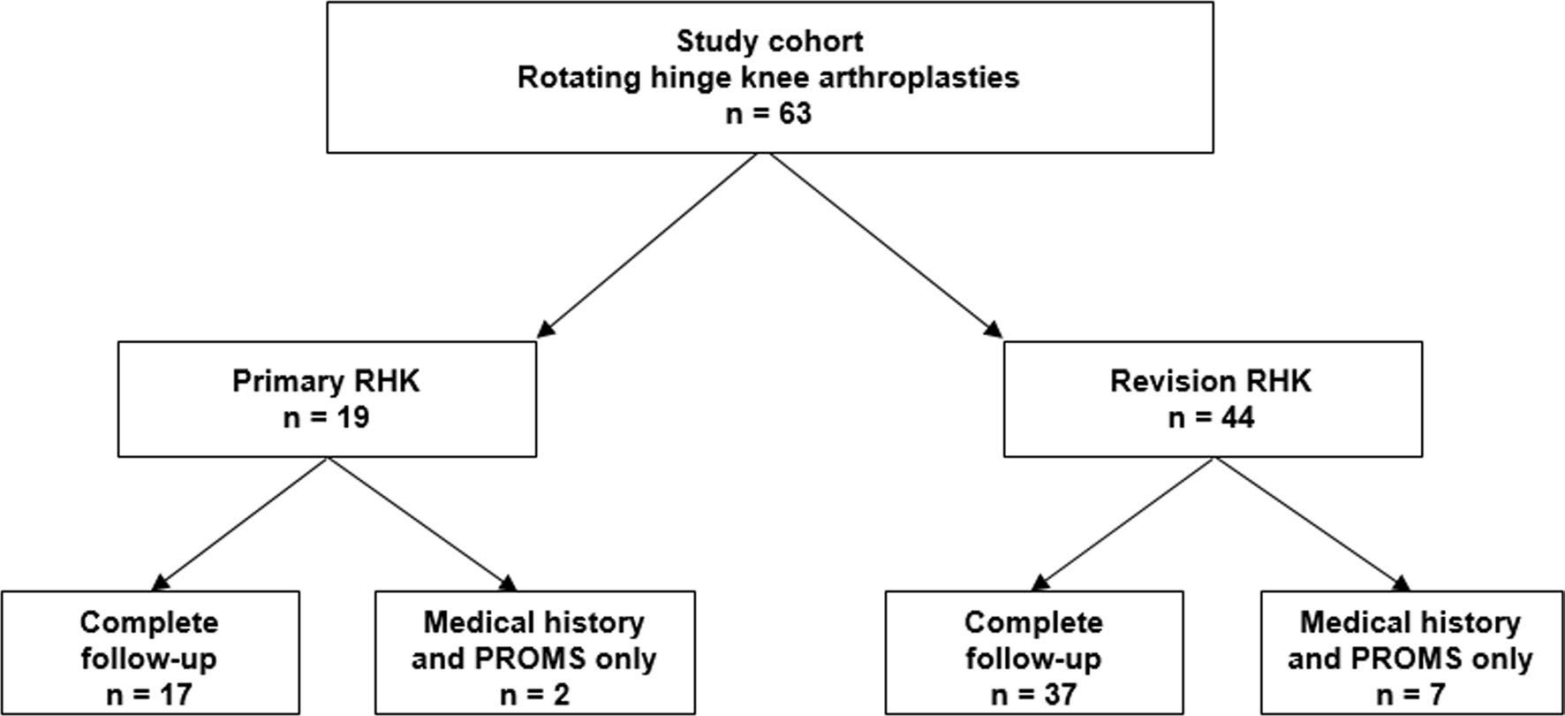
Flowchart demonstrating study cohort and kind of follow-up

Nineteen RHKs were implanted as primary TKA and 44 in revision cases. A flowchart illustrating the study cohort is provided in Fig. 1. Prior to revision surgery a primary TKA (*n* = 31), a condylar constrained TKA (*n* = 9), a hinged prosthesis (*n* = 3), and a uni-condylar prosthesis (*n* = 1) had been used. In the primary group in 17 cases, a complete follow-up with clinical and radiological evaluation as well as PROMs and analysis of the medical history was possible. Two patients did not present at our outpatient clinic and therefore only medical history and PROMs could be obtained. In the revision group, 37 cases were available for complete and seven for partial follow-up (without clinical and radiological assessment).

The radiologic assessment consisted of an antero-posterior (a.p.) and lateral radiograph of the knee as well as a standing long leg radiograph. Clinical examination focused on the evaluation of instability and range of motion. Moreover, Hospital of Special Surgery Score (HSS), Knee Society Score (KSS), Oxford Knee Score (OKS), EQ-5D-3L, and Visual Analog Scale (VAS) for overall function and satisfaction were raised. The outcome ranges of the used scores are reported in Table 1.

**Table 1:** Range of the used scores

Score	Sub-score	Worst possible score	Best possible score
HSS score		4	100
	HSS objective	0	48
	HSS symptoms	4	52
KSS objective score		2	105
	KSS objective	0	80
	KSS symptoms	2	25
KSS expectation and satisfaction score		3	55
	KSS satisfaction	0	40
	KSS expectation	3	15
KSS functional activity score		0	100
	KSS walking and standing	0	30
	KSS standard activities	0	30
	KSS advanced activities	0	25
	KSS discretionary activities	0	15
OKS score (low score is better)		60	12
VAS for overall function		0	100
EQ-5D Index		0	1

*HSS* Hospital for Special Surgery score, *KSS* Knee Society Score, *OKS* Oxford Knee Score, *VAS* Visual Analog Scale, *EQ-5D* measure of health-related quality of life

The radiographs were analyzed for signs of implant loosening defined as a gap of more than 2 mm in the bone-cement- or bone-implant-interface [16]. The anatomical axis was measured on the long leg radiographs. The medical history, including the surgical report were evaluated for the number of previous surgeries, the decision making for a RHK and subsequent revisions. Furthermore, demographic data including patient age, ASA-Score, height, and body weight were collected.

### Statistical analysis

Statistical analysis was conducted in SPSS (IBM SPSS Statistics, Version 25 for Windows) and STATA 16.1 (StataCorp, The College Station, Texas, USA) with a significance level of *p* < 0.05. The Kolmogorov–Smirnov test was used to check for normal distribution of data. Data is reported as mean ± standard deviation (SD). An unpaired t-test was used to compare the outcome in primary and revision cases. All variables (BMI and sex) that showed at least a very weak evidence (*p* < 0.2) for an association with the exposure (primary or revision arthroplasty), were included in a multivariable linear regression analyzed for the different outcome parameters. No adjustment of multiple testing was performed. Kaplan–Meier survival graphs were calculated for the endpoints revision and implant survival.

## Results

The main indications for revision that lead to the use of a RHK were infection (*n* = 16) and aseptic loosening (*n* = 15) followed by instability (*n* = 8), painful TKA without a specific reason (*n* = 4) and polyethylene wear (*n* = 1).

The main reasons to use the RHK as a primary implant were severe medial and/or lateral ligamentous instability (*n* = 15) and fixed valgus deformity over 20 degrees (*n* = 12) followed by extension deficit over 20 degrees (*n* = 7), rheumatoid arthritis with severe bone destruction (*n* = 5), and posttraumatic bone loss (*n* = 2). All patients in this group possessed two or more of the above-mentioned pathologies.

At the final follow-up limited medio-lateral instability of less than 5 mm was detected in 7 cases (13%). In the other 47 cases (87%) no instability was present. An antero-posterior instability was not detected in this cohort. The mean range of motion was 115 ± 17 degrees.

PROMS showed satisfactory results in the upper third of each scale and are displayed in Table 2.

**Table 2:** Demographic, radiological and functional data of all included patients

Gender	Female	59% (37/63)
	Male	41% (26/63)
Age at index surgery (years)		69 ± 9 (63)
ASA classification	ASA 1	3% (2/63)
	ASA 2	29% (18/63)
	ASA 3	64% (40/63)
	ASA 4	5% (3/63)
BMI (kg/m^2^)		30 ± 6 (63)
Affected side	Right	60% (38/63)
	Left	40% (25/63)
Kind of follow-up		
	Complete follow-up	86% (54/63)
	Scores and medical history	14% (9/63)
Kind of surgery		
	Primary	30% (19/63)
	First Revision	44% (28/63)
	Second Revision	13% (8/63)
	Third revision	11% (7/63)
	Forth revision	2% (1/63)
Reasons for implantation in revision cases		
	Loosening	34% (15/44)
	Infection	36% (16/44)
	Wear	2% (1/44)
	Instability	18% (8/44)
	Painful TKA without obvious reason	9% (4/44)
Reasons for implantation in primary cases		
	Excessive valgus	63% (12/19)
	Rheumatoid arthritis	26% (5/19)
	Ligamentous instability	79% (15/19)
	Bone loss	11% (2/19)
	Extension deficit	37% (7/19)
Status before revision with RHK		
	Standard prosthesis (CR, PS, etc.)	71% (31/44)
	Condylar constrained Prosthesis	20% (9/44)
	Hinged prosthesis	7% (3/44)
	Uni-condylar prosthesis	2% (1/44)
Mean follow-up (months)		34 ± 8 (63)
Reasons for revision		
	Infection	100% (4/4)
Time to revision (months)		6 ± 5 (4)
Kind of first revision		
	DAIR	75% (3/4)
	2-stage-exchange	25% (1/4)
*Radiological outcome*		
Varus axis (°)		7 (1)
Valgus axis (°)		6 ± 2 (53)
Loosening (radiolucent line > 2 mm)		9% (5/54)
*Functional outcome*		
ROM (°)		115 ± 17 (54)
Medio-lateral stability		
	No instability	87% (47/54)
	Instability < 5 mm	13% (7/54)
	Instability > 5 mm	0% (0/54)
*Antero-posterior stability*		
	No instability	100% (54/54)
HSS score		79 ± 18 (54)
	HSS objective	43 ± 4 (54)
	HSS symptoms	43 ± 8 (63)
KSS objective score		78 ± 27 (63)
	KSS objective	70 ± 9 (54)
	KSS symptoms	18 ± 6 (63)
KSS expectation and satisfaction score		37 ± 11 (63)
	KSS satisfaction	28 ± 9 (63)
	KSS expectation	10 ± 3 (63)
KSS functional activity score		55 ± 25 (63)
	KSS walking and standing	20 ± 10 (63)
	KSS standard activities	19 ± 7 (63)
	KSS advanced activities	9 ± 7 (63)
	KSS discretionary activities	7 ± 5 (63)
OKS (12 = best result, 60 = worst result)		26 ± 10 (63)
VAS for overall function and satisfaction		70 ± 20 (63)
EQ-5D index (Reference: German value set)		0.741 ± 0.233 (63)

Abbreviations: *ASA* American society of anesthesiologists, *BMI* body mass index, *TKA* total knee arthroplasty, *RHK* rotating hinge knee, *ROM* range of motion. *HSS* Hospital for Special Surgery score. *KSS* Knee Society Score. *OKS* Oxford Knee Score. *VAS* Visual Analog Scale. *EQ-5D* measure of health-related quality of life

In 53 cases a mean anatomical valgus of 6 ± 2 degrees was measured, in one case a varus malalignment of 7 degrees was present. In five cases a radiolucent line of at least 2 mm width suspicious for implant loosening was detected in the bone-cement or bone-implant interface of tibia and/or femur. Four of those patients were without clinical symptoms. One patient experienced pain during walking but refused to undergo revision for aseptic loosening.

Subgroup analysis comparing the outcome of RHK implantation in primary and revision cases, revealed a significantly better outcome in the primary group with regards to ROM (*p* = 0.002), HSS (*p* = 0.035), KSS expectation and satisfaction score (*p* = 0.004), KSS functional activity score (*p* = 0.023), and OKS (*p* = 0.047). KSS objective score (*p* = 0.070), VAS for overall function and satisfaction (*p* = 0.117), and the EQ-5D index (*p* = 0.377) did not show significant differences. After including gender and BMI as possible confounding factors via multivariate linear regression, significant differences between primary and revision group could only be detected for ROM (*p* = 0.004), KSS expectation and satisfaction scores (*p* = 0.030) and the KSS satisfaction sub-score (*p* = 0.035). The results are as well summarized and reported in Tables 2 and 3.

**Table 3:** Demographic, radiological and functional comparison of primary versus revision cases

		Primary	Revision	*p*	*p* (multivariate linear regression)
*N*		19	44		
Gender	Female	42% (8/19)	66% (29/44)		
	Male	58% (11/19)	34% (15/44)		
Age at index surgery (years)		68 ± 8 (19)	70 ± 9 (4)	.502	
ASA classification	ASA 1	5% (1/19)	2% (1/44)		
	ASA 2	37% (7/19)	25% (1/44)		
	ASA 3	58% (11/19)	66% (29/44)		
	ASA 4	0% (0/19)	7% (3/44)		
BMI (kg/m^2^)		26 ± 5 (19)	32 ± 6 (19)	.001*	
Affected side	Right	68% (13/19)	57% (25/44)		
	Left	32% (6/19)	43% (19/44)		
Kind of follow-up					
	Complete follow-up	90% (17/19)	84% (37/44)		
	Scores and medical history	10% (2/19)	16% (7/44)		
Mean follow-Up (months)		32 ± 6 (19)	35 ± 8 (44)	.135	
Reasons for revision					
	Infection	5% (1/19)	7% (3/44)		
Time to revision (months)		1 (1/1)	8 ± 5 (3)		
Kind of first revision					
	DAIR	100% (1/1)	67% (2/3)		
	2-stage-exchange	0% (0/1)	33% (1/3)		
*Radiological outcome*					
Varus axis (°)		- (0)	7 (1)		
Valgus axis (°)		7 ± 3 (17)	6 ± 2 (36)	.074	
Loosening (radiolucent line > 2 mm)		0% (0/17)	14% (5/37)		
*Functional outcome*					
ROM (°)		126 ± 12 (17)	111 ± 17 (37)	.002*	.004*
Medio-lateral stability					
	No instability	100% (17/17)	81% (30/37)		
	Instability < 5 mm	0% (0/17)	19% (7/37)		
	Instability > 5 mm	0% (0/17)	0% (0/37)		
Antero-posterior stability					
	No instability	100% (17/17)	100% (37/37)		
HSS score		87 ± 19 (19)	76 ± 17 (44)	.035*	.127
	HSS objective	45 ± 3 (17)	42 ± 4 (37)	.005*	.105
	HSS symptoms	47 ± 8 (19)	41 ± 8 (44)	.025*	.104
KSS objective score		87 ± 26 (19)	74 ± 27 (44)	.070	.133
	KSS objective	73 ± 9 (17)	68 ± 8 (37)	.048*	.168
	KSS symptoms	22 ± 4 (19)	16 ± 7 (44)	.002*	.008
KSS expectation and satisfaction score		44 ± 9 (19)	35 ± 11 (44)	.004*	.030*
	KSS satisfaction	32 ± 7 (19)	26 ± 9 (44)	.006*	.035*
	KSS expectation	11 ± 3 (19)	9 ± 3 (44)	.023*	.108
KSS functional activity Score		66 ± 28 (19)	50 ± 23 (44)	.023*	.223
	KSS walking and standing	22 ± 10 (19)	18 ± 10 (44)	.086	.565
	KSS standard activities	23 ± 8 (19)	18 ± 7 (44)	.013*	.111
	KSS advanced activities	12 ± 8 (19)	7 ± 5 (44)	.007*	.083
	KSS discretionary activities	8 ± 6 (19)	7 ± 4 (44)	.382	.740
OKS (12 = best result, 60 = worst result)		22 ± 11 (19)	28 ± 9 (44)	.047*	.254
VAS for overall function and satisfaction		77 ± 17 (19)	68 ± 23 (44)	.117	.358
EQ-5D index (Reference: German value set)		0.781 ± 0.265 (19)	0.724 ± 0.219 (44)	.377	.940

Abbreviations: *ASA* American society of anesthesiologists, *BMI* body mass index, *DAIR* debridement and implant retention, *TKA* total knee arthroplasty, *RHK* rotating hinge knee, *ROM* range of motion, *HSS* Hospital for Special Surgery score, *KSS* Knee Society Score, *OKS* Oxford Knee Score, *VAS* Visual Analog Scale, *EQ-5D* measure of health-related quality of life, Significance-level: *p* < .05, Significant values are marked (*)

**Table 4:** Outcome of rotating hinge TKA – a literature comparison

Study	Number of cases	Usage in primary or revision cases	Mean follow-up	KSS Score	HSS Score	OKS Score	Survival rate	Reoperation rate
Kocaoğlu et al. 2022	15	Revision	8 years	NA	NA	NA	80% (at 5 years) 64% (at 10 years)	40%
Wignadasan et al. 2021	41	Revision	14 years	NA	NA	41	90%	NA
Hintze et al. 2021	27	Primary	7 years	NA	NA	38	95% (at 10 years)	8%
Kendoff et al. 2020	160	Primary	14 years	NA	NA	NA	88% (at 14 years)	18%
Neri et al. 2020	112	Primary	7 years	64	NA	33	91%	28%
Abdulkarim et al. 2019 (Review)	1425	Primary and revision	NA	85	NA	NA	92% (at five years) 82% (at 10 years)	NA
Rouquette	40	Primary and revision	18 months	NA	NA	NA	95%	20%
Kouk et al. 2018 (Review)	NA	Primary and revision	NA	NA	NA	NA	51%-93% (at 10 years)	NA
Cottino et al.2017	408	Primary and revision	4 years	81	NA	NA	90% (at 2 years) 77% (at 10 years)	11%
Baker et al. 2014	46	Primary	7 years			32	97% (at 5 years)	NA
Gudnason et al. 2011	42	Revision	9 years	85	67	NA	65% (at 10 years)	NA

*HSS* Hospital for Special Surgery score, *KSS* Knee Society Score, *OKS* Oxford Knee Score, *NA* Not available

**Fig. 2:**
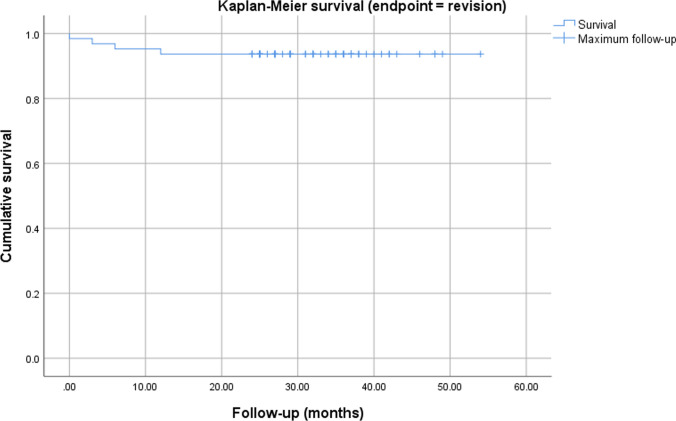
Kaplan–Meier survival chart with endpoint revision. The * x*-axis shows the maximum follow-up, while the * y*-axis indicates the survival with endpoint revision

**Fig. 3:**
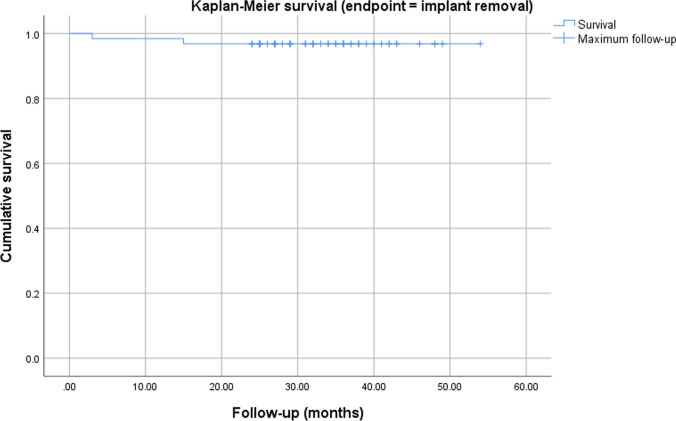
Kaplan–Meier survival chart with endpoint implant removal. The * x*-axis shows the maximum follow-up, while the * y*-axis indicates the survival with endpoint implant removal

At the final follow-up four cases had been revised (6.3%) and in two cases (3.2%) the implant had been removed, resulting in an implant survival of 96.8% at a mean follow-up of 34 months. The Kaplan–Meier survival graphs for the endpoints revision and implant removal are displayed in Figs. 2 and 3. One infection was considered chronic and was treated with a two-stage-exchange. In the other three cases a debridement, change of the mobile parts and administration of antibiotics (DAIR procedure) was performed due to acute infection. The mean time to revision was 6 ± 5 months. The closer analysis of these cases revealed one early infection (three weeks after implantation due to wound healing problems) in a primary case that was treated successfully with DAIR. The other three cases were in the revision group and the initial RHK implantation had already been for infection. Two of them were treated with DAIR and one with a two-stage-exchange. One prosthesis of the latter group that received DAIR in the first place had to undergo one-stage-revision three months later due to persistent infection.

## Discussion

Rotating hinge knees are important implants to address anatomic deficiencies like ligamentous instability, bone loss, and gross deformities that cannot be treated with standard TKA or even higher constrained but not hinged implant. The primary application of rotating hinge implants are complex TKA revision surgeries. [14, 25] RHKs are associated with a higher complication rate as compared to standard implants but the results improved during the last 20 years [18]. These implants are necessary and valuable tools in cases where standard implants reach their limits, but the indication should be strict due to higher complication and revision rates. The most frequent conditions leading to the implantation of a RHK in this study were infection and aseptic loosening, followed by ligamentous instability. This is in accordance with the available literature. [4, 8, 25]

The reason for using such implants in primary cases is the complex nature of these cases including severe bone loss, high grade deformities, ligamentous and muscular insufficiency associated with an increased risk of a less favorable outcome [21]. Another proposed indication is a intraarticular fracture in the elderly patient [19]. First generation hinged implants showed high rates of mechanical complications due to their high degree of constraint. [21] Even current generation RHKs such as the one used in this cohort were shown to be associated with high revision rates of 10–20% during the first 2 years when used in revision and complex primary cases. [7, 8, 22, 23] There is an ongoing debate on, whether RHK should be used in complex primary cases. [9] Recent studies showed acceptable survival rates of 80–90% after 7–10 years. [1, 15, 20] Therefore, there is a trend to a recommendation of this implants in complex primary situations. Nevertheless, age and indications might be important factors influencing survival. A recent study of 238 primary RKH with a minimum follow-up of 10 years, showed a survival of 94% in the age group over 60 years, while below that age only 77% survival could be reached. Moreover, the same study found a survival of 96% in varus knees and of 79% in valgus knees. The combination of age under 60 and valgus even worsened the survival to 64% [12]. These significant differences in survival rates should sensitize the surgeon to make patient selection for primary RHK very carefully. The average age of 68 years in our cohort may be one of the reasons for the good survival. A condylar constrained knee should be thought of as an alternative which has been reported to have significantly better outcomes compared to RHK [17].

Regarding clinical outcome, the here reported KSS score of 78 points shows slightly inferior outcome compared to primary standard TKA. A study of primary TKA with standard implants reported a KSS score of 92 points at a medium-term follow-up of 6.9 years [3]. Considering the longer follow-up, our values are slightly inferior, but still striking given the complex primary situation. Unfortunately, there is no exclusive study of complex cases treated with standard implants that would allow a direct comparison of our results. The mean outcome scores in a study reporting outcomes of a RHI with comparable follow-up were mean 81 points in the KSS objective score and 36 points in the KSS functional score. [4] The respective values in our study were 78 point in the KSS objective score, which is in the same range, while the KSS functional score was better in our cohort with 55 points.

Looking at the EQ-5D VAS level for overall function and satisfaction, no representative data was available for Switzerland. Therefore, we compared our data to the biggest neighbor country Germany, where a mean EQ-5D VAS score for overall function and satisfaction of 69 has been reported in the age group of 65–74 years [11]. This cohort data represents a cross section of society without elaborating on preexisting conditions or prior surgery. The value of 70 points given here corresponds to this representative reference population. When comparing primary and revision cases, the former showed significantly better results. Though after multivariate linear regression comparable functional outcomes in almost every (sub-) score were found.

After a mean follow-up of 3 years, implant survival rate was 97%, which is remarkable as 70% of the implants were used in revision cases. An exclusive study of one-stage revision using an RHK implant showed a revision rate of 40% at 8 years postoperatively with an implant survival of 80% at five years postoperative [13]. These markedly inferior results compared to our cohort highlight the in the literature available discrepancy regarding outcomes of RHK. In comparison, a study on 408 RHK reported a revision rate of 9.7% at two years postoperatively which better matches our revision rate of 6.3% at three years postoperative [4]. Table 4 gives an overview on outcome and survival rate of relevant studies reporting on rotating hinge implants.

Due to its retrospective character, our study has some limitations. First, preoperative PROMS were not available, making it impossible to evaluate disability before surgery or indicate improvement with surgery. This circumstance complicates the comparability of our data with the literature. Second, only 30% of the cases were primary cases, leading to statistical inaccuracy while comparing primary and revision cases. Third, the mean follow-up of 3 years is too short to give general recommendations for this type of implant especially when used as a primary implant. Forth, the different gender and BMI distribution were possible confounders with the result that only ROM and satisfaction were significantly lower in the revision group, while no differences could be detected for the other outcome parameters. Fifth, the number of cases was too small to further assess and compare the outcomes with regard to the indication leading to primary implantation or revision. However, there are only few studies available evaluating the survival and functional outcome of modern RHKs. The present study is the first one reporting the survival and outcome of this specific implant (GMK Hinge, Medacta international, Castel San Pietro, Switzerland). The case number is limited but within the range of other studies reporting on such rather rarely used implants [1]. The follow-up of other studies evaluating RHK ranges from 1 to 17 years with the majority of studies reporting short- to medium-term follow-up [14].

## Conclusions

Rotating hinge knee arthroplasty can be performed with good clinical outcome and low complication rate in revision and complex primary cases. The latter is associated with even better mid-term results. RHK is an option in cases where standard arthroplasty and even implants with a higher degree of constraint have reached their limits. The increasing use of these implants will also provide a basis for reliable long-term outcome data.
